# Plasma membrane-associated cation-binding protein 1-like protein negatively regulates intercellular movement of BaMV

**DOI:** 10.1093/jxb/erx307

**Published:** 2017-08-29

**Authors:** Ying-Ping Huang, Ying-Wen Huang, I-Hsuan Chen, Lin-Ling Shenkwen, Yau-Huei Hsu, Ching-Hsiu Tsai

**Affiliations:** 1 Graduate Institute of Biotechnology, National Chung Hsing University, Taichung, 402, Taiwan; 2 Research Center for Sustainable Energy and Nanotechnology, National Chung Hsing University, Taichung, 402, Taiwan

**Keywords:** Bamboo mosaic virus, defense protein, NbPCaP1L, *Nicotiana benthamiana*, positive-sense RNA virus, replicase, viral RNA movement

## Abstract

To establish a successful infection, a virus needs to replicate and move cell-to-cell efficiently. We investigated whether one of the genes upregulated in *Nicotiana benthamiana* after Bamboo mosaic virus (BaMV) inoculation was involved in regulating virus movement. We revealed the gene to be a plasma membrane-associated cation-binding protein 1-like protein, designated *NbPCaP1L*. The expression of *NbPCaP1L* in *N. benthamiana* was knocked down using Tobacco rattle virus-based gene silencing and consequently the accumulation of BaMV increased significantly to that of control plants. Further analysis indicated no significant difference in the accumulation of BaMV in *NbPCaP1L* knockdown and control protoplasts, suggesting *NbPCaP1L* may affect cell-to-cell movement of BaMV. Using a viral vector expressing green fluorescent protein in the knockdown plants, the mean area of viral focus, as determined by fluorescence, was found to be larger in *NbPCaP1L* knockdown plants. Orange fluorescence protein (OFP)-fused NbPCaP1L, NbPCaP1L-OFP, was expressed in *N. benthamiana* and reduced the accumulation of BaMV to 46%. To reveal the possible interaction of viral protein with NbPCaP1L, we performed yeast two-hybrid and co-immunoprecipitation experiments. The results indicated that NbPCaP1L interacted with BaMV replicase. The results also suggested that NbPCaP1L could trap the BaMV movement RNP complex via interaction with the viral replicase in the complex and so restricted viral cell-to-cell movement.

## Teaser

Plasma membrane-associated cation-binding protein 1-like protein of *Nicotiana benthamiana* plays a role in defense against Bamboo mosaic virus by binding the viral replicase and restricting viral intercellular movement.

## Introduction

To complete an infection cycle, a plant virus needs to not only replicate efficiently but also move cell-to-cell successfully in the form of a virion or viral ribonucleoprotein complex (RNP) via the plasmodesmata (PD) (Hofmann *et al.*, 2007). The latter process requires both viral-encoded movement proteins (MPs) and host factors (Scholthof, 2005; Taliansky *et al.*, 2008; Benitez-Alfonso *et al.*, 2010; Niehl and Heinlein, 2011; Schoelz *et al.*, 2011). In addition, the cytoskeleton system is involved in transporting the viral components to the PD (Prokhnevsky *et al.*, 2005; Avisar *et al.*, 2008). Although the microtubules do not play a major role in viral RNA/protein complex trafficking in tobacco mosaic virus (TMV), it is still a key player involved in delivering the complex to microfilaments for transportation (Li *et al.*, 2011; Peña and Heinlein, 2012; Liu and Nelson, 2013). Potato mop-top virus (PMTV) possesses a triple gene block (TGB) that encodes three MPs for virus movement. Mutants with an N-terminal 84-amino acid deletion of TGB1 in PMTV, with lost activity in microtubule association, failed to show long distance movement (Agre *et al.*, 1998). A host protein NbMPB2Cb from *N. benthamiana*, a homolog of TMV-MP30 binding protein 2C, MPB2C, which is involved in organizing the cortical microtubules (Chaumont *et al.*, 2000), could regulate the movement of Potato virus X (PVX). The movement of PVX is restricted when NbMPB2Cb is overexpressed; by contrast the movement is enhanced when the expression of NbMPB2Cb is silenced (Hachez *et al.*, 2013).

Bamboo mosaic virus (BaMV) is a member of the *Potexvirus* genus of *α-Flexiviridae*. The genome of BaMV has one single-stranded positive-sense RNA approximately 6.4 kb long with a 5′ m^7^GpppG structure and 3′ adenylates numbering ~250−300 ( Lin *et al.*, 1992; Chen *et al.*, 2005). The genomic RNA of BaMV contains five open reading frames, ORFs 1 to 5. ORF 1 encodes a 155 kDa polypeptide comprising three functional domains. The N-terminal capping enzyme domain possesses S-adenosylmethionine–dependent guanylytransferase activity (Li *et al.*, 2001*a*; Huang *et al.*, 2004; Huang *et al.*, 2005); the middle part is a helicase-like domain with nucleoside triphosphatase (NTPase) and RNA 5′-triphosphatase activities (Li *et al.*, 2001*b*) and the C-terminal RNA-dependent RNA polymerase (RdRp) domain (Li *et al.*, 1998; Huang *et al.*, 2001) has viral RNA replication activity. The overlapped ORFs 2 to 4, TGBp1 to 3, encode MPs for cell-to-cell movement (Lin *et al.*, 2004; Lin *et al.*, 2006). ORF 5 encodes a 25 kDa viral capsid protein (CP) required for virion assembly, symptom development, and movement in plant cells (Lan *et al.*, 2010; Hung *et al.*, 2014a; Hung *et al.*, 2014b). Infected protoplasts and plants show two major subgenomic RNAs, 2 and 1 kb long (Tsai *et al.*, 1999; Yeh *et al.*, 1999).

Several host factors positively or negatively regulate the infection cycle of BaMV. The chloroplast phosphoglycerate kinase (Cheng *et al.*, 2013a), heat-shock protein 90 (Huang *et al.*, 2012), a glutathione transferase NbGSTU4 (Chen *et al.*, 2013), an exoribonuclease NbXRN4 (Lee *et al.*, 2015), and a Rab-GTPase NbRABG3f (Huang *et al.*, 2016) are involved in assisting viral RNA replication. A Ser/Thr kinase-like protein NbSTKL (Cheng *et al.*, 2013b) and a Rab-GTPase activation protein NbRabGAP1 (Huang *et al.*, 2013) support viral movement. By contrast, a cytoplasmic form of glyceraldehyde 3-phosphate dehydrogenase (Prasanth *et al.*, 2011) and a putative methyltransferase (Cheng *et al.*, 2009) suppress BaMV RNA replication by interacting with the 3′ UTR and replicase, respectively.

A group of proteins on the plasma membrane containing six transmembrane domains termed plasma membrane intrinsic proteins (PIPs) play roles in regulating the diffusion of water and small uncharged solutes (Agre *et al.*, 1998; Schäffner, 1998; Chaumont *et al.*, 2000; Hachez *et al.*, 2013). Some PIPs can even transport hydrogen peroxide in response to stresses (Jang *et al.*, 2012). A new class of PIPs, without any cross-membrane domain and associated with the plasma membrane via myristoylation, bind Ca^2+^, Mg^2+^, and Cu^2+^ and are called plasma membrane-associated cation-binding proteins (PCaPs) (Ide *et al.*, 2007; Nagasaki-Takeuchi *et al.*, 2008). Arabidopsis PCaP1 incorporates [^3^H]myristic acid during *in vitro* transcription and translation and interacts with phosphatidylinositol phosphates (PtdInsPs). Furthermore, AtPCaP1 could bind calmodulin in a Ca^2+^-dependent manner (Nagasaki *et al.*, 2008). Hence, AtPCaP1 could be involved in intracellular signaling via interaction with PtdInsPs and calmodulin (Kato *et al.*, 2010).

The 25 kDa protein AtPCaP1 is also called microtubule-destabilizing protein 25 (MDP25) and was found to regulate the elongation of hypocotyl cells by destabilizing cortical microtubules dependent on calcium (Li *et al.*, 2011). When cytoplasmic calcium increases, MDP25 binds directly to and destabilizes microtubules to enhance its depolymerization (Qin *et al.*, 2012). In addition, AtPCaP1 can interact with the movement-required novel protein P3N-PIPO of Turnip mosaic virus (TuMV) and promote viral cell-to-cell movement (Vijayapalani *et al.*, 2012). Disrupted expression of AtPCaP1 reduced the accumulation and cell-to-cell movement of TuMV, which led to enhanced plant resistance.

In this study, we investigated one gene with upregulated expression from a previous study of BaMV inoculation (Cheng *et al.*, 2010) that may play a negative role in regulating the accumulation of BaMV. This gene, designated *NbPCaP1L*, is an ortholog of *PCaP1* in *N. benthamiana*. BaMV accumulation was increased when *NbCaP1L* expression was knocked down but was decreased when *NbCaP1L* was transiently overexpressed. Further analysis indicated that NbCaP1L acts on the movement of BaMV. The mechanism of this regulation is discussed.

## Materials and methods

### Viruses and plants

BaMV, PVX, and Cucumber mosaic virus (CMV) were used for inoculation. *N. benthamiana* were grown at 28°C in a growth room with a 16 h light/8 h dark cycle.

### Virus particle purification

The infected leaves were homogenized with extraction buffer of 0.5 M borate at pH 9.0, 1 mM EDTA, and 0.1% β-mercaptoethanol, in a 1 g/2 ml ratio, filtrated through a miracloth, and centrifuged at 12000×g at 4°C for 10 min. The supernatant was stirred at 4℃ for 10 min with the addition of 4M K_2_HPO_4_ and 2M CaCl_2_ to 1% and 2%, respectively, and was centrifuged at 12000×g at 4°C for 10 min. The supernatant was stirred at 4℃ for 30 min with the addition of triton X-100 and PEG 6000 to 2% and 6%, respectively, and was centrifuged at 12000×g at 4°C for 10 min. The pellet was resuspended in BE buffer of 50 mM borate at pH 8.0 and 1 mM EDTA, and centrifuged at 14300×g at 4°C for 1 h on a cushion of 20 % sucrose. The virus pellet was resuspended in 10 ml BE buffer and quantification carried out using a spectrophotometer.

### Virus-induced gene silencing (VIGS) and virus challenge

Tobacco rattle virus (TRV)-based VIGS was used to knock down the expression of *NbPCaP1L*. The cDNA fragment ACGT11 derived from NbPCaP1L was obtained from cDNA-AFLP and cloned into pGEM-T Easy vector (Promega, Madison, WI, USA) previously (Cheng *et al.*, 2010). The 118 bp cDNA was isolated by digestion with *EcoR*I and subcloned into the pTRV2 vector. The resulting plasmid was electroporated into the *Agrobacterium tumefaciens* C58C1 for knockdown experiments as described previously (Huang *et al.*, 2013). Approximately 500 ng of viral particles was mechanically inoculated into the fourth leaf above the infiltrated leaves at 10 d post-infiltration (dpi). Total proteins were extracted from these virus-inoculated leaves at 1, 3, 5, and 7 dpi with BaMV inoculation and 5 dpi with CMV and PVX inoculation.

### Cloning the NbPCaP1L gene

To clone the full-length *NbPCaP1L* gene, the 3′ primer (5′ *CTCGAG*TCAGTCG ACAGCTTTTGGTGGTTCCGGT 3′) and 5′ primer (5′ T*GCTAGC GGATCC*AT GATGGGTTATTGGCAAGC 3′) were used for PCR. The 3′ primer contains the *Xho*I site (underlined) and the 5′ primer contains the *Nhe*I and *BamH*I sites (underlined) for cloning. Full-length *NbPCaP1L* was cloned into the pGEM-T Easy vector (Promega). The sequence was verified by sequencing on an IR^2^ System (LI-COR Biosciences, Lincoln, NE, USA). Finally, the full-length *NbPCaP1L* gene was subcloned into the pBIN-OFP vector containing orange fluorescent protein (OFP) driven by the CMV 35S promoter (reconstructed from pmKO2-S1; MBL international, Woburn, MA, USA).

### Protoplast isolation and inoculation

Protoplasts were isolated from the fourth leaf above *N. benthamiana* leaves infiltrated with TRV/Luc, containing part of the luciferase gene from nucleotide 110 to 508, and TRV/*NbPCaP1L* at 10 d post Agro-infiltration. The detailed protocol for protoplast isolation was previously described (Tsai *et al.*, 1999). In brief, approximately 4 g of tissue was digested with 25 ml of enzyme solution at 25°C overnight. Intact mesophyll protoplasts were collected from the interphase of the 0.55 M mannitol-MES solution and the 0.55 M sucrose cushion. Finally, protoplasts were washed and re-suspended in mannitol-MES solution. Approximately 2.5 × 10^5^ protoplasts were inoculated with 1 µg BaMV RNA with 20% polyethyleneglycol-6000. The inoculated protoplasts were incubated at 25°C under constant light. Total protein and RNA were extracted from protoplasts after 24 h of incubation.

### Western blot assay

Total protein harvested from inoculated protoplasts or leaves was separated in 12% SDS-PAGE and transferred to nitrocellulose membranes (PROTRAN BA 85 Schleicher & Schnell), which were probed with the rabbit antibodies for BaMV, PVX, or CMV CP, then incubated with IRDye 800-conjugated affinity-purified anti-rabbit IgG antibody (Rockland Immunochemicals, Gilbertsville, PA, USA). The fluorescence density on membranes was determined using LI-COR Odyssey (LI-COR Biosciences).

### RNA extraction and real-time quantitative RT-PCR

Inoculated leaves of *NbPCaP1L* knockdown *N. benthamiana* were collected and ground. Total RNA was extracted as described (Lin *et al.*, 2007). First-strand cDNA was synthesized with 20 pmole 39d(T) oligo primer and reverse transcriptase (Promega) according to the manufacturer’s protocol. Real-time quantitative PCR was performed in a 20 μl-reaction containing a 1000X dilution of SYBR green I (Cambrex Bio Science Rockland, ME, USA) with primer GT11_5′ (5′- AAGGTTGTTCCAAAATTAAAGC-3′) and GT11_3′ (5′- TTCAATCCTGAAACCTTTGGTCCC-3′) in 0.2-ml PCR tubes. The conditions began with an initial hold at 95°C for 5 min, followed by 30 cycles of 94°C for 30 sec, 56°C for 30 sec and 72°C for 30 sec. The expression of β-actin was amplified with the primer pair actin_5′ (5′ GATGAAGATACTCACAGAAAGA 3′) and actin_3′ (5′ GTGGTTTCATGAATGCCAGCA 3′) as an internal control for normalization.

### Detection of BaMV cell-to-cell movement

Approximately 10 d after VIGS infiltrated with TRV/*luciferase* or TRV/*NbPCaP1L*, the fourth and fifth leaves above infiltrated leaves of *N. benthamiana* plants were mechanically inoculated with 7.5 μg pCBG (Lin *et al.*, 2006), the infectious cDNA clone carrying the green fluorescent protein (GFP) gene driven by the BaMV subgenomic promoter. The fluorescence derived from GFP accompanied by BaMV movement was captured by inverted fluorescent microscopy (Olympus IX71) with an excitation wavelength of 460 to 495 nm, an emission wavelength of 510 nm, and a dichromatic mirror at 505 nm at 6 dpi. The area of each lesion was measured using Image J (http://rsbweb.nih.gov/ij/).

### Laser scanning confocal microscopy

Plasmids pBIN-OFP (vector only) and pBIN-NbPCaP1L-OFP were transformed into *A. tumefaciens* C58C1. *A. tumefaciens* containing pBIN-OFP, pBIN-NbPCaP1L-OFP or pBIN61-HcPro was cultured to OD600=1; and the cells were resuspended in 10 mM MgCl_2_ with 500 μM acetosyringone for induction after being spun down. The pBIN-OFP or pBIN-NbPCaP1L culture was mixed with that of pBIN61-HcPro in a 1:1 volume ratio and infiltrated into *N. benthamiana* leaves. The images were taken by laser scanning confocal microscopy (Olympus Fluoview FV1000) with 405 nm and 543 nm laser excitation for cyan fluorescent protein and OFP, respectively, 2 d after infiltration.

### Yeast two-hybrid interaction

The gene fragments encoding the MP and CP of BaMV were constructed into the prey plasmid pYESTrp2 and designated pYES-TGBp1, -2, -3, and -CP. The replication-related DNA fragments were constructed into the bait plasmid pHybLex/Zeo and designated pLEX-Capping, -RdRp, and -Helicase (Cheng *et al.*, 2009; Lee *et al.*, 2011). The DNA fragment for cloning *NbPCaP1L* into yeast prey and bait plasmids was amplified with two sets of primers: for the prey plasmid, YES5′PCaP1HindIII (5′ GGG*AAGCTT*ATGATGGGTTATTGGCAAGC 3′) and YES3′PCaP1XhoI (5′ *GTCGAC*TCAGTCGACAGCTTTTG GTGGTTCCGGT 3′), and for the bait plasmid, LEX5′P CaP1EcoRI (5′ *GAATTC*ATGATGGGTTATTGGCAAGC 3′) and LEX3′PCaP1PstI (5′ G*CTGCAG*TCAGTCG ACAGCTTTTGGTGGTTCCGGT 3′) (restriction enzyme site underlined). The amplified DNAs were cloned into pGEM-T Easy vector (Promega) and verified by sequencing. The full-length *NbPCaP1L* was then subcloned into the yeast prey plasmid with *Hin*dIII and *Xho*I sites and into the bait plasmid with *Eco*RI and *Pst*I sites. *Saccharomyces cerevisiae* strain L40, harboring the bait plasmid (pLEX-Capping, -RdRp, or -Helicase) or prey plasmid (pYES-TGBp1, -2, -3, and -CP), was transformed with the prey plasmid pYES-NbPCaP1L or bait plasmid pLEX-NbPCaP1L, respectively. Positive colonies were grown on Trp^-^/His^-^/Zeocin selection agar plates.

### Co-immunoprecipitation

*N. benthamiana* leaves were infiltrated with the Agrobacteria mixture containing the plasmids pKBRepHA21 plus pEpyon-OFP or pKBRepHA21 plus pBIN-NbPCaP1L-OFP. Total proteins were extracted using an extraction buffer of 20 mM Tris–HCl at pH 7.5, 2 mM MgCl_2_, 300 mM NaCl, 5 mM DTT, and 1% protease inhibitor cocktail (Roche), at 3 dpi. The supernatant was collected after centrifugation at 4000×g at 4°C for 10 min and underwent immunoprecipitation with anti-HA antibody (Sigma-Aldrich, H9658) at 4°C for 4 h. The interacting proteins were incubated with 20 μl protein A magnetic beads (GE Healthcare) at 4°C for 2 h. Finally, the samples were washed, eluted, and underwent western blot analysis with anti-HA (Sigma-Aldrich, H6908), anti-OFP, or anti-BaMV CP antibodies.

## Results

### Cloning the plasma membrane–associated cation-binding protein 1-like gene NbPCaP1L from *N. benthamiana*

A cDNA fragment *ACGT11* 118 bp in length was found upregulated after BaMV inoculation by cDNA-AFLP and was revealed to have an effect on BaMV accumulation in *N. benthamiana* (Cheng *et al.*, 2010). This 118 bp cDNA fragment had 94% identity to that of PIP from *N. tabacum* (GenBank accession Y08609.1). This PIP was defined as a new family of PIP because it lacks a transmembrane domain, whereas conventional PIPs have six membrane-spanning domains with cytosolic N- and C-termini (Lin *et al.*, 2011; Zhao *et al.*, 2013). In a further analysis, PIP from *N. tabacum* showed 57% identity to the PCaP1 of *Arabidopsis thaliana* (GenBank accession NM_118145) (see Supplementary Fig. S1 at *JXB* online). The identity of this PIP from *N. tabacum* is more similar to PCaP1 than conventional PIP.

To clone the full-length *ACGT11* from *N. benthamiana*, we designed primers according to the conserved region among the PCaP1 orthologs from different species (Supplementary Fig. S1) and the nucleotide sequence of PIP from *N. tabacum.* The coding sequence of the PCaP1-like gene from *N. benthamiana*, designated *NbPCaP1L* (GenBank accession MF346700), is 711 nucleotides long encoding a 236-amino acid polypeptide and has 89% and 88% nucleotide and amino acid identity to *NtPIP* (Supplementary Fig. S1).

### Reducing the expression of NbPCaP1L increases BaMV accumulation in *N. benthamiana* plants but not protoplasts

**Fig. 1.**
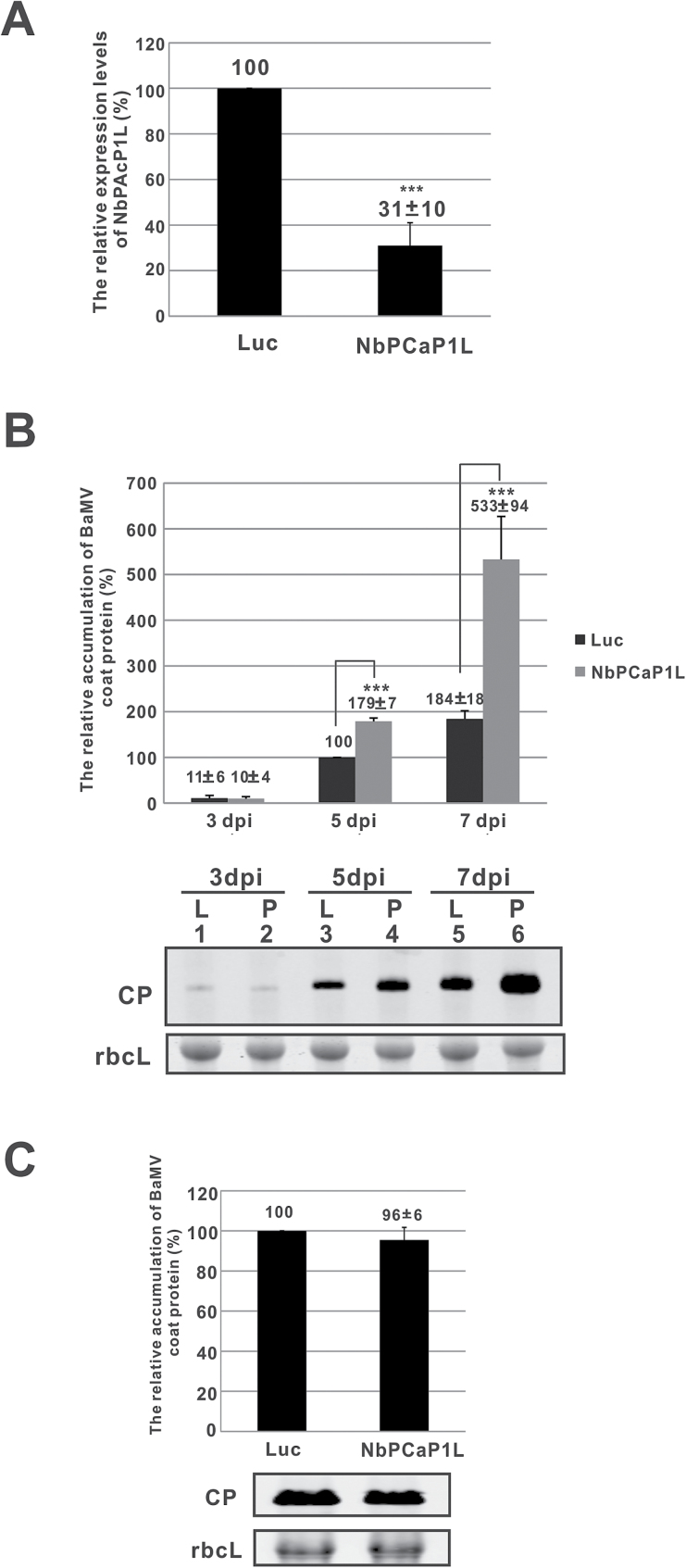
The relative accumulation of BaMV coat protein in *NbPCaP1L* knockdown plants and protoplasts. (A) The expression of *NbPCaP1L* mRNA was determined by real-time quantitative RT-PCR in *Luc* and *NbPCaP1L* knockdown leaves. (B) Western blot analysis of coat protein (CP) accumulation on the inoculated leaves. The accumulation of control plants was set to 100%. Data are mean ± standard deviation relative levels of BaMV CP from at least three independent experiments with at least three plants in each experiment. Representative western blot results of CP levels with triplicate results and rbcL as a control. (C) Western blot analysis of CP accumulation. The accumulation in control protoplasts at 24 h post inoculation (hpi) was set to 100%. Representative results are shown. Data are mean ± standard deviation relative levels of BaMV CP from at least three independent experiments. Luc, luciferase knockdown control plants or protoplasts; NbPCaP1L, NbPCaP1L knockdown plants or protoplasts; CP, BaMV coat protein; rbcL, RuBisCO large subunit (the loading control for normalization). **P<0.01, ***P<0.001 by Student’s *t*-test.

To characterize the role of *NbPCaP1L* in BaMV infection, we used TRV-based VIGS to knock down the expression of *NbPCaP1L* and inoculate BaMV. Knockdown of *NbPCaP1L* expression by 70% compared with control plants, *Luc*-knockdown plants, as determined by real-time qRT-PCR (Fig. 1A), conferred no morphologic alterations (Supplementary Fig. S2). After normalization with the large subunit of RuBisCO stained with Coomassie blue, western blot analysis revealed that BaMV CP levels increased in *NbPCaP1L* knockdown plants to 180% that of control plants at 5 dpi but not at earlier infection times (Fig. 1B). Furthermore, BaMV levels were strikingly increased up to 2.9-fold in *NbPCaP1L* knockdown plants at 7 dpi (Fig. 1B). Hence, *NbPCaP1L* may suppress the accumulation of BaMV CP in plants, especially during late infection.

To investigate whether increased BaMV CP accumulation in *NbPCaP1L* knockdown plants was caused by enhanced viral RNA replication or viral movement in plants, we prepared *NbPCaP1L* knockdown protoplasts to exclude the movement effect. *NbPCaP1L* knockdown and control protoplasts showed no difference in BaMV accumulation (Fig. 1C). Hence, reducing the expression of *NbPCaP1L* did not impede the replication process of BaMV RNA. *NbPCaP1L* could be involved in regulating BaMV cell-to-cell movement. To determine whether reduced *NbPCaP1L* expression could affect other viruses, we inoculated *NbPCaP1L* knockdown plants with PVX and CMV. *NbPCaP1L* knockdown and control plants did not differ in CP accumulation of these viruses at 5 dpi (Supplementary Fig. S3). The effect of *NbPCaP1L* on viral accumulation may be specific to BaMV but not PVX or CMV.

### NbPCaP1L regulates BaMV cell-to-cell movement

**Fig. 2.**
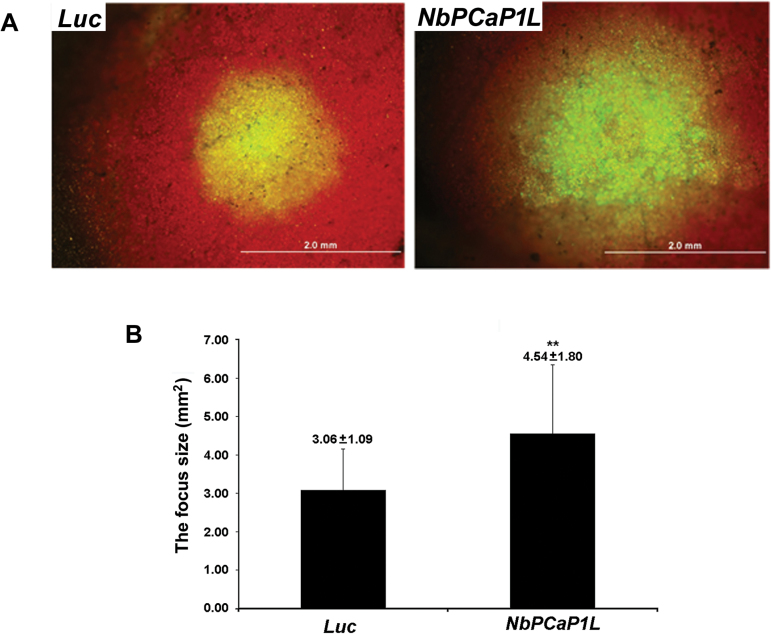
Cell-to-cell movement of BaMV in *Luc* and *NbPCaP1L* knockdown plants. (A) Fluorescent microscopy of the area of fluorescent foci in inoculated leaves of luciferase knockdown control (*Luc*) and *NbPCaP1L* knockdown (*NbPCaP1L*) plants after inoculation with the BaMV infectious plasmid carrying green fluorescent protein (GFP). Scale bar, 2 mm. (B) Statistical analysis. Data are mean ± standard deviation of 19 and 29 foci from *Luc* and *NbPCaP1L* knockdown plants, respectively. **P<0.01 by Student’s *t*-test.

As the results of the *NbPCaP1L* knockdown experiments indicated that *NbPCaP1L* had no effect on BaMV accumulation in protoplasts but a marked effect in plants, *NbPCaP1L* may be involved in BaMV cell-to-cell movement. To test this, *NbPCaP1L*- and *Luc*-knockdown plants were inoculated with the full-length infectious BaMV cDNA plasmid pCBG expressing GFP with the BaMV subgenomic promoter (Lin *et al.*, 2004), with measurements taken at 6 dpi (Fig. 2A). The mean GFP focus area in *Luc* and *NbPCaP1L* knockdown plants was 3.06 ± 1.09 and 4.54 ± 1.80 mm^2^, respectively (Fig. 2B). Reducing the expression of *NbPCaP1L* in *N. benthamiana* plants made them more accessible to BaMV infection, with more and larger foci than in control plants.

### BaMV accumulation is reduced in NbPCaP1L- expressing leaves

**Fig. 3.**
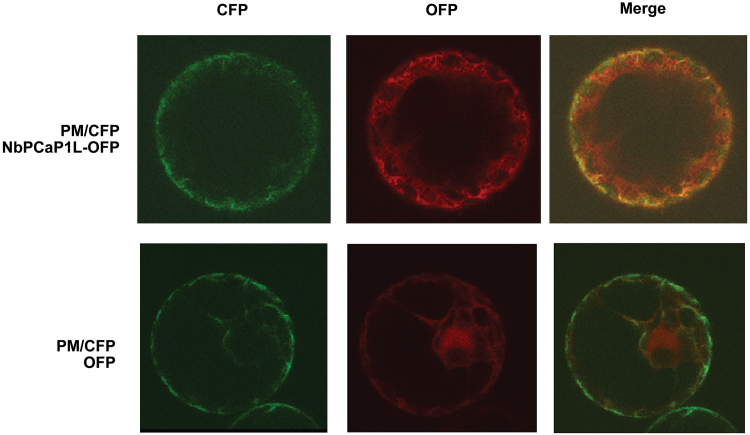
The localization at the plasma membrane (PM) and cytoplasm of NbPCaP1L with OFP fused at the C-terminus and transiently expressed in *N. benthamiana* protoplasts. Cyan fluorescence protein (CFP) fused with a plasma membrane aquaporin AtPIP2A is transiently expressed in protoplasts and is used as the PM marker.

**Fig. 4.**
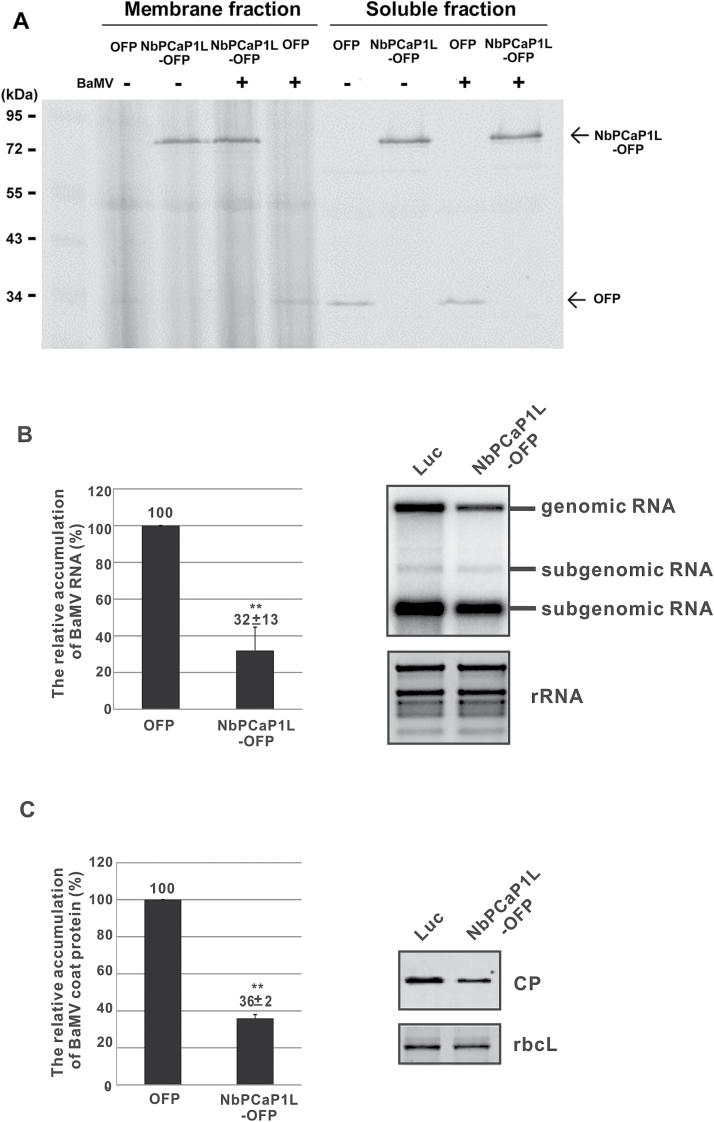
Effect of expression of *NbPCaP1L* on BaMV infection. (A) Western blot analysis of the fractionation of transiently expressed NbPCaP1L-OFP and OFP alone. Relative accumulation of BaMV CP (B) and RNAs (C) at 5 d post-inoculation (dpi) on inoculated leaves, with NbPCaP1L-OFP or OFP only (as a control) transiently expressed on the same leaves at 3 dpi. Data are mean ± standard error of at least three independent experiments. **P<0.01 by Student’s *t*-test.

To visualize the subcellular localization of NbPCaP1L protein in cells, we fused OFP at the C-terminus of NbPCaP1L and transiently expressed it in *N. benthamiana* plants. NbPCaP1L-OFP was clearly present in both the plasma membrane, as it co-localized with a plasma membrane marker, and cytoplasm in protoplasts (Fig. 3). Further analysis of protein extracts after fractionation revealed that NbPCaP1L-OFP was equally distributed in the membrane and soluble fractions. This distribution profile of NbPCaP1L-OFP in *N. benthamiana* cells in the membrane and soluble fraction was not changed after BaMV infection (Fig. 4A). The subcellular localization of AtPCaP1 was reported to target the cytoskeleton, cell junction, plasmodesmata, and cell membrane via lipid-anchoring (Nühse *et al.*, 2003; Marmagne *et al.*, 2004; Ide *et al.*, 2007; Nagasaki *et al.*, 2008; Li *et al.*, 2011; Vijayapalani *et al.*, 2012). AtPCaP1 shuttled from the plasma membrane to cytoplasm with increased calcium levels (Kato *et al.*, 2010; Li *et al.*, 2011). The subcellular localization of NbPCaP1L-OFP in both the cytoplasm and plasma membrane was therefore similar to that of AtPCaP1.

To validate whether NbPCaP1L plays a negative role in BaMV infection, we inoculated NbPCaP1L-OFP transiently expressing leaves with BaMV. The BaMV RNA and CP levels were significantly reduced to 32% and 36% of that of control plants, respectively (Fig. 4B, C). Hence, NbPCaP1L negatively regulates BaMV movement.

### NbPCaP1L interacts with BaMV replicase

**Fig. 5.**
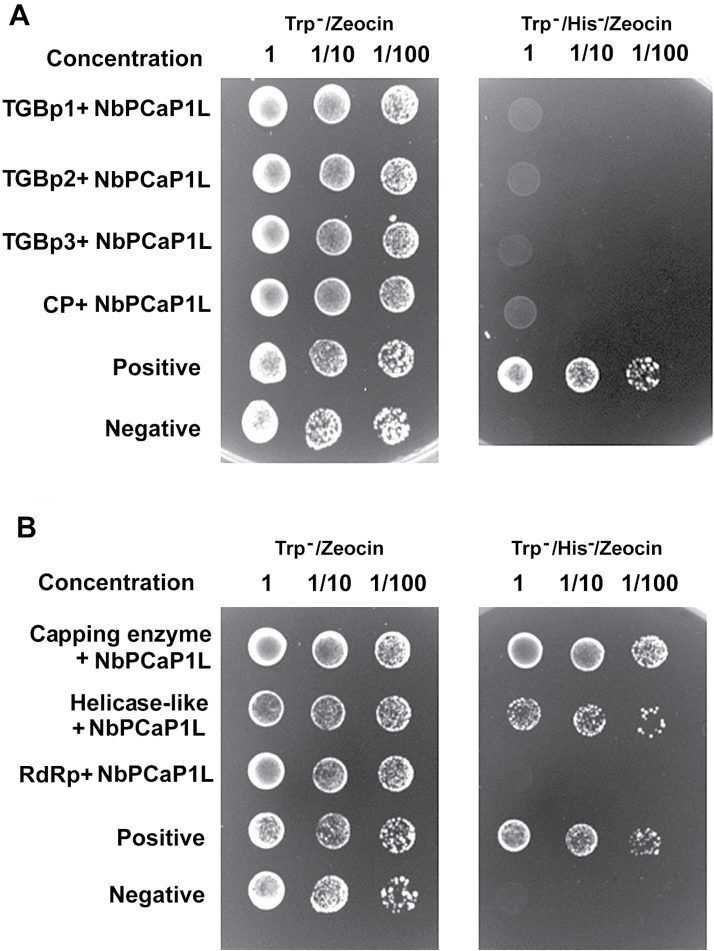
Interaction of NbPCaP1L with BaMV-encoded polypeptides in yeast cells. Yeast strain L40 co-transformed with the prep plasmid, pYES-TGBp1, -2, -3, or -CP, and bait plasmid pLEX-NbPCaP1L in (A) or prey plasmid pYES-NbPCaP1L and bait plasmid, pLEX-Capping, -RdRp, or -Helicase in (B). Positive control, yeast containing pLEX-Fos2 and pYES-Jun; negative control, yeast containing pHybLex/Zeo and pYESTrp2. Positive colonies were grown on Trp^-^/His^-^/Zeocin selection agar plates. The yeast concentrations with the dilution factor are indicated at the top of each panel.

To examine whether the host protein NbPCaP1L interacts with any viral-encoded proteins, we used the yeast two-hybrid system. The genes encoding the MPs and CP of BaMV were constructed in a yeast prey plasmid; full-length NbPCaP1L was cloned into the bait plasmid and transformed into yeast containing each of the viral-encoding genes, TGBp1, 2, 3, and CP. No yeast could grow on the selective medium (Trp^-^/His^-^/Zeocin) except the positive control yeast (Fig. 5A).

Another set of constructs containing each domain of the BaMV-encoded replicase, namely the capping, helicase-like, and RdRp domains, was cloned into the bait plasmid of the yeast two-hybrid system (Cheng *et al.*, 2009; Lee *et al.*, 2011). NbPCaP1L was then constructed into the prey plasmid for testing of the interaction with BaMV replicase. The capping and helicase-like domains could interact with NbPCaP1L (Fig. 5B). Overall, these results suggest that the host protein NbPCaP1L could interact with the N-terminal portion of BaMV replicase, that is the capping and helicase-like domains, in the yeast cells.

**Fig. 6.**
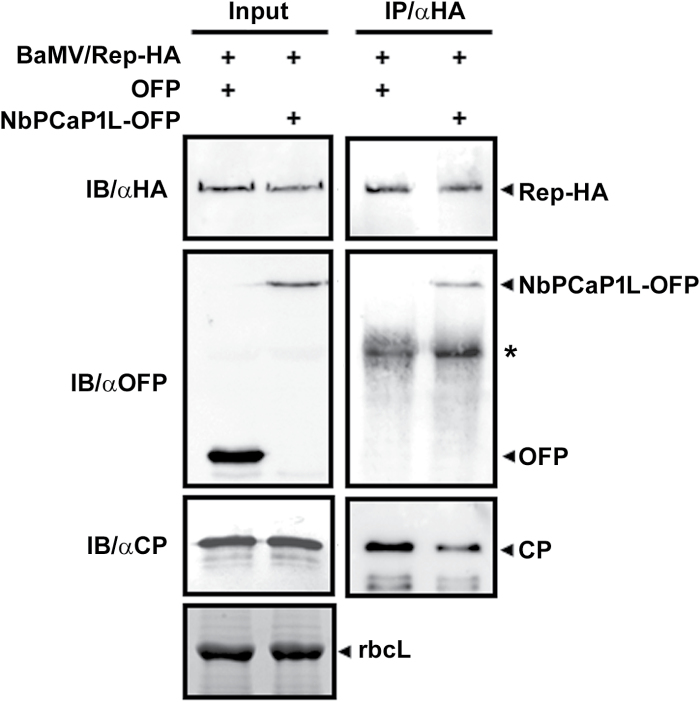
Co-immunoprecipitation (IP) assay to verify the interaction between BaMV replication protein and NbPCaP1L. Total proteins (input) were extracted from *N. benthamiana* leaves co-infiltrated with Agrobacteria containing the infectious BaMV/Rep-HA and OFP vector or NbPCaP1L-OFP. Equal amounts of input proteins were used for immunoblotting (IB) with antibodies against HA, OFP, and BaMV CP as indicated. rbcL, RuBisCo large subunit is used as the loading control. After IP with anti-HA antibody, the co-IP of Rep-HA, NbPCaP1L-OFP, and CP was detected using corresponding antibodies for IB.

To validate the results of the yeast two-hybrid finding that NbPCaP1L interacts with BaMV replicase, we used co-immunoprecipitation. BaMV modified with a HA-tag at the C-terminus of the replicase, BaMV/Rep-HA, was agro-infiltrated onto transiently expressed OFP or NbPCaP1L-OFP leaves. Total proteins were immunoprecipitated with anti-HA antibody. NbPCaP1L-OFP could interact with replicase-HA but not OFP alone (Fig. 6).

## Discussion

The process of viral movement is critical for plant viruses to establish a successful infection, even with effective replication in cells. Here we investigated whether one host gene from *N. benthamiana*, a plasma membrane-associated cation-binding protein 1-like protein designated *NbPCaP1L*, which shows upregulated expression after BaMV inoculation, was involved in regulating virus movement.

Plants have evolved a few different strategies, such as post-transcriptional gene silencing (Hammond *et al.*, 2001) and hypersensitive response (Baurès *et al.*, 2008), to protect themselves against pathogen infection. However, pathogens can also develop a counter-strike mechanism to break down the host defense systems, such as virus-encoded silencing suppressors. The host could therefore have evolved some other unidentified defense mechanisms to resist the pathogen infection. These types of offense/defense strategies must be identified to gain knowledge into plant protection, especially for important crops.

These unidentified defense genes might express their genes differentially during pathogen infection. We previously used cDNA-AFLP to isolate the differentially expressed genes from *N. benthamiana* plants after BaMV infection (Cheng *et al.*, 2010). *NbPCaP1L* was one of the potential upregulated defense genes based on our results in this study, which could act on viral movement.

The movement complex of potexviruses, including PVX and BaMV, was proposed to assemble on the perinuclear endoplasmic reticulum-derived membrane-bound body (Tian *et al.*, 2004; Verchot-Lubicz *et al.*, 2010). The complex, comprising the membrane-associated TGBp2 and TGBp3 translated at the endoplasmic reticulum (Verchot-Lubicz *et al.*, 2010) and ribonucleoparticles containing viral RNA, CP, TGBp1, and replicase (Tian *et al.*, 2004; Lee *et al.*, 2011; Wu *et al.*, 2011; Park *et al.*, 2013), was transported to the endoplasmic reticulum network directly or to actin filaments associated with membrane vesicles toward the PD (Tian *et al.*, 2004). The cellular vesicle formation factor Rab-GTPase activation protein, (NbRabGAP1, regulates BaMV cell-to-cell and systemic movement (Huang *et al.*, 2013), so the membrane vesicle could be involved in trafficking the BaMV movement complex.

The association of viral replicase in the movement complex was first proposed by observing the differential time required for the spread of TMV from primary inoculated cells to secondary cells, at 18 to 20 h post inoculation, and secondary cells to tertiary cells, as 2 to 4 h post inoculation (Kawakami *et al.*, 2004). Viral RNA in primary inoculated cells was suggested to need more time to set up the movement complex for trafficking to secondary cells, whereas the movement complex containing the replicase could initiate replication on entry to secondary cells and speed up the assembly of the movement complex to tertiary cells. The interaction between BaMV CP and replicase is needed for efficient viral movement. Mutations in CP that fail to interact with viral replicase diminish cell-to-cell movement in plants (Lee *et al.*, 2011). The anti-TGBp3 immunopurified movement complex from BaMV-infected *N. benthamiana* harbors endogenous RdRp activity (Chou *et al.*, 2013), so BaMV replicase may be included in the movement complex.

The host protein NbPCaP1L could interact with the replicase of BaMV (Figs 5 and 6), so the mechanism of NbPCaP1L retarding the movement of BaMV could involve competing for replicase availability with the competent viral movement complex. Although we did not observe the interaction between NbPCaP1L with viral membrane-associated MPs TGBp2 and TGBp3 in the yeast two-hybrid system (Fig. 5), which might not be a good way to detect this interaction, NbPCaP1L constraining viral movement via interaction with MPs cannot be ruled out. We did however demonstrate that the interaction of NbPCaP1L with BaMV replicase could be the major effector for restricting virus movement.

Arabidopsis PCaP1 could assist the viral movement of TuMV via interaction with the potyviral protein P3N-PIPO of the viral movement complex to target the plasma membrane and localize to the plasmadesmata (Vijayapalani *et al.*, 2012). By contrast, the homolog protein NbPCaP1L in *N. benthamiana* for BaMV may play a different role in retarding viral movement by interacting with the replication protein in the movement complex. NbPCaP1L is a membrane-associated protein that harbors a novel activity by interacting with the viral RdRp and trapping the viral movement complex to restrict viral cell-to-cell movement.

## Supplementary data

Supplementary data are available at *JXB* online.

Fig. S1. Sequence alignment and the expression profile of NbPCaP1L after BaMV inoculation.

Fig. S2. The morphology of control and *NbPCaP1L* knockdown plants.

Fig. S3. The relative accumulation of viral coat protein (CP) in *NbPCaP1L*-knockdown and control plants.

## Supplementary Material

Supplementary_figures_S1_S3

## Abbreviations:

BaMV: bamboo mosaic virus
CMV: cucumber mosaic virus
CP: capsid protein
dpi: days post infiltration
GFP: green fluorescent protein
MPs: movement proteins
OFP: orange fluorescent protein
PCaPs: plasma membrane-associated cation-binding proteins
PD: plasmodesmata
PIPs: plasma membrane intrinsic proteins
PMTV: potato mop-top virus
PVX: potato virus X
RdRp: RNA-dependent RNA polymerase
RNP: ribonucleoprotein complex
TGB: triple gene block
TMV: tobacco mosaic virus
TRV: tobacco rattle virus
TuMV: Turnip mosaic virus
VIGS: virus-induced gene silencing.

## References

[CIT0001] Agre P, Bonhivers M, Borgnia MJ 1998 The aquaporins, blueprints for cellular plumbing systems. The Journal of Biological Chemistry 273, 14659–14662. 9614059 10.1074/jbc.273.24.14659

[CIT0002] Avisar D, Prokhnevsky AI, Dolja VV 2008 Class VIII myosins are required for plasmodesmatal localization of a closterovirus Hsp70 homolog. Journal of Virology 82, 2836–2843. 18199648 10.1128/JVI.02246-07 PMC2258991

[CIT0003] Baurès I, Candresse T, Leveau A, Bendahmane A, Sturbois B 2008 The Rx gene confers resistance to a range of potexviruses in transgenic *Nicotiana* plants. Molecular Plant-Microbe Interactions 21, 1154–1164. 18700820 10.1094/MPMI-21-9-1154

[CIT0004] Benitez-Alfonso Y, Faulkner C, Ritzenthaler C, Maule AJ 2010 Plasmodesmata: gateways to local and systemic virus infection. Molecular Plant-Microbe Interactions 23, 1403–1412. 20687788 10.1094/MPMI-05-10-0116

[CIT0005] Chaumont F, Barrieu F, Jung R, Chrispeels MJ 2000 Plasma membrane intrinsic proteins from maize cluster in two sequence subgroups with differential aquaporin activity. Plant Physiology 122, 1025–1034. 10759498 10.1104/pp.122.4.1025 PMC58937

[CIT0006] Chen IH, Chiu MH, Cheng SF, Hsu YH, Tsai CH 2013 The glutathione transferase of *Nicotiana benthamiana* NbGSTU4 plays a role in regulating the early replication of *Bamboo mosaic virus*. New Phytologist 199, 749–757. 23701112 10.1111/nph.12304 PMC3744755

[CIT0007] Chen IH, Chou WJ, Lee PY, Hsu YH, Tsai CH 2005 The AAUAAA motif of *Bamboo mosaic virus* RNA is involved in minus-strand RNA synthesis and plus-strand RNA polyadenylation. Journal of Virology 79, 14555–14561. 16282455 10.1128/JVI.79.23.14555-14561.2005 PMC1287560

[CIT0008] Cheng CW, Hsiao YY, Wu HC 2009 Suppression of bamboo mosaic virus accumulation by a putative methyltransferase in *Nicotiana benthamiana*. Journal of Virology 83, 5796–5805. 19297487 10.1128/JVI.02471-08 PMC2681968

[CIT0009] Cheng SF, Huang YP, Chen LH, Hsu YH, Tsai CH 2013 Chloroplast phosphoglycerate kinase is involved in the targeting of *Bamboo mosaic virus* to chloroplasts in *Nicotiana benthamiana* plants. Plant Physiology 163, 1598–1608. 24154620 10.1104/pp.113.229666 PMC3846135

[CIT0010] Cheng SF, Huang YP, Wu ZR, Hu CC, Hsu YH, Tsai CH 2010 Identification of differentially expressed genes induced by *Bamboo mosaic virus* infection in *Nicotiana benthamiana* by cDNA-amplified fragment length polymorphism. BMC Plant Biology 10, 286. 21184690 10.1186/1471-2229-10-286 PMC3024324

[CIT0011] Cheng SF, Tsai MS, Huang CL, Huang YP, Chen IH, Lin NS, Hsu YH, Tsai CH, Cheng CP 2013 Ser/Thr kinase-like protein of *Nicotiana benthamiana* is involved in the cell-to-cell movement of *Bamboo mosaic virus*. PLoS ONE 8, e62907. 23646157 10.1371/journal.pone.0062907 PMC3639906

[CIT0012] Chou YL, Hung YJ, Tseng YH 2013 The stable association of virion with the triple-gene-block protein 3-based complex of *Bamboo mosaic virus*. PLoS Pathogens 9, e1003405. 23754943 10.1371/journal.ppat.1003405 PMC3675025

[CIT0013] Hachez C, Besserer A, Chevalier AS, Chaumont F 2013 Insights into plant plasma membrane aquaporin trafficking. Trends in Plant Science 18, 344–352. 23291163 10.1016/j.tplants.2012.12.003

[CIT0014] Hammond SM, Caudy AA, Hannon GJ 2001 Post-transcriptional gene silencing by double-stranded RNA. Nature Reviews. Genetics 2, 110–119. 10.1038/35052556 11253050

[CIT0015] Hofmann C, Sambade A, Heinlein M 2007 Plasmodesmata and intercellular transport of viral RNA. Biochemical Society Transactions 35, 142–145. 17233621 10.1042/BST0350142

[CIT0016] Huang CY, Huang YL, Meng M, Hsu YH, Tsai CH 2001 Sequences at the 3’ untranslated region of *Bamboo mosaic potexvirus* RNA interact with the viral RNA-dependent RNA polymerase. Journal of Virology 75, 2818–2824. 11222706 10.1128/JVI.75.6.2818-2824.2001 PMC115907

[CIT0017] Huang YL, Han YT, Chang YT, Hsu YH, Meng M 2004 Critical residues for GTP methylation and formation of the covalent m7GMP-enzyme intermediate in the capping enzyme domain of *Bamboo mosaic virus*. Journal of Virology 78, 1271–1280. 14722282 10.1128/JVI.78.3.1271-1280.2004 PMC321370

[CIT0018] Huang YL, Hsu YH, Han YT, Meng M 2005 mRNA guanylation catalyzed by the S-adenosylmethionine-dependent guanylyltransferase of *Bamboo mosaic virus*. The Journal of Biological Chemistry 280, 13153–13162. 15677480 10.1074/jbc.M412619200

[CIT0019] Huang YP, Chen JS, Hsu YH, Tsai CH 2013 A putative Rab-GTPase activation protein from *Nicotiana benthamiana* is important for *Bamboo mosaic virus* intercellular movement. Virology 447, 292–299. 24210126 10.1016/j.virol.2013.09.021

[CIT0020] Huang YP, Jhuo JH, Tsai MS, Tsai CH, Chen HC, Lin NS, Hsu YH, Cheng CP 2016 NbRABG3f, a member of Rab GTPase, is involved in *Bamboo mosaic virus* infection in *Nicotiana benthamiana*. Molecular Plant Pathology 17, 714–726. 26416342 10.1111/mpp.12325 PMC6638505

[CIT0021] Huang YW, Hu CC, Liou MR, Chang BY, Tsai CH, Meng M, Lin NS, Hsu YH 2012 Hsp90 interacts specifically with viral RNA and differentially regulates replication initiation of *Bamboo mosaic virus* and associated satellite RNA. PLoS Pathogens 8, e1002726. 22654666 10.1371/journal.ppat.1002726 PMC3359997

[CIT0022] Hung CJ, Hu CC, Lin NS, Lee YC, Meng M, Tsai CH, Hsu YH 2014 Two key arginine residues in the coat protein of *Bamboo mosaic virus* differentially affect the accumulation of viral genomic and subgenomic RNAs. Molecular Plant Pathology 15, 196–210. 24393453 10.1111/mpp.12080 PMC6638855

[CIT0023] Hung CJ, Huang YW, Liou MR, Lee YC, Lin NS, Meng M, Tsai CH, Hu CC, Hsu YH 2014 Phosphorylation of coat protein by protein kinase CK2 regulates cell-to-cell movement of *Bamboo mosaic virus* through modulating RNA binding. Molecular Plant-Microbe Interactions 27, 1211–1225. 25025779 10.1094/MPMI-04-14-0112-R

[CIT0024] Ide Y, Nagasaki N, Tomioka R, Suito M, Kamiya T, Maeshima M 2007 Molecular properties of a novel, hydrophilic cation-binding protein associated with the plasma membrane. Journal of Experimental Botany 58, 1173–1183. 17264065 10.1093/jxb/erl284

[CIT0025] Jang JY, Rhee JY, Chung GC, Kang H 2012 Aquaporin as a membrane transporter of hydrogen peroxide in plant response to stresses. Plant Signaling & Behavior 7, 1180–1181. 22899075 10.4161/psb.21178 PMC3489655

[CIT0026] Kato M, Nagasaki-Takeuchi N, Ide Y, Tomioka R, Maeshima M 2010 PCaPs, possible regulators of PtdInsP signals on plasma membrane. Plant Signaling & Behavior 5, 848–850. 20448467 10.4161/psb.5.7.11825 PMC3014536

[CIT0027] Kawakami S, Watanabe Y, Beachy RN 2004 *Tobacco mosaic virus* infection spreads cell to cell as intact replication complexes. Proceedings of the National Academy of Sciences, USA 101, 6291–6296. 10.1073/pnas.0401221101 PMC395962 15079061

[CIT0028] Lan P, Yeh WB, Tsai CW, Lin NS 2010 A unique glycine-rich motif at the N-terminal region of *Bamboo mosaic virus* coat protein is required for symptom expression. Molecular Plant-Microbe Interactions 23, 903–914. 20521953 10.1094/MPMI-23-7-0903

[CIT0029] Lee CC, Ho YN, Hu RH, Yen YT, Wang ZC, Lee YC, Hsu YH, Meng M 2011 The interaction between *Bamboo mosaic virus* replication protein and coat protein is critical for virus movement in plant hosts. Journal of Virology 85, 12022–12031. 21917973 10.1128/JVI.05595-11 PMC3209275

[CIT0030] Lee CC, Lin TL, Lin JW, Han YT, Huang YT, Hsu YH, Meng M 2015 Promotion of *Bamboo mosaic virus* accumulation in *Nicotiana benthamiana* by 5’→3’ exonuclease NbXRN4. Frontiers in Microbiology 6, 1508. 26779163 10.3389/fmicb.2015.01508 PMC4702010

[CIT0031] Li J, Wang X, Qin T 2011 MDP25, a novel calcium regulatory protein, mediates hypocotyl cell elongation by destabilizing cortical microtubules in *Arabidopsis*. The Plant Cell 23, 4411–4427. 22209764 10.1105/tpc.111.092684 PMC3269874

[CIT0032] Li YI, Chen YJ, Hsu YH, Meng M 2001a Characterization of the AdoMet-dependent guanylyltransferase activity that is associated with the N terminus of *Bamboo mosaic virus* replicase. Journal of Virology 75, 782–788. 11134291 10.1128/JVI.75.2.782-788.2001 PMC113974

[CIT0033] Li YI, Cheng YM, Huang YL, Tsai CH, Hsu YH, Meng M 1998 Identification and characterization of the *Escherichia coli*-expressed RNA-dependent RNA polymerase of *Bamboo mosaic virus*. Journal of Virology 72, 10093–10099. 9811749 10.1128/jvi.72.12.10093-10099.1998 PMC110542

[CIT0034] Li YI, Shih TW, Hsu YH, Han YT, Huang YL, Meng M 2001b The helicase-like domain of plant potexvirus replicase participates in formation of RNA 5’ cap structure by exhibiting RNA 5’-triphosphatase activity. Journal of Virology 75, 12114–12120. 11711602 10.1128/JVI.75.24.12114-12120.2001 PMC116107

[CIT0035] Lin JW, Ding MP, Hsu YH, Tsai CH 2007 Chloroplast phosphoglycerate kinase, a gluconeogenetic enzyme, is required for efficient accumulation of *Bamboo mosaic virus*. Nucleic Acids Research 35, 424–432. 17169994 10.1093/nar/gkl1061 PMC1802604

[CIT0036] Lin L, Luo Z, Yan F, Lu Y, Zheng H, Chen J 2011 Interaction between potyvirus P3 and ribulose-1,5-bisphosphate carboxylase/oxygenase (RubisCO) of host plants. Virus Genes 43, 90–92. 21400205 10.1007/s11262-011-0596-6

[CIT0037] Lin MK, Chang BY, Liao JT, Lin NS, Hsu YH 2004 Arg-16 and Arg-21 in the N-terminal region of the triple-gene-block protein 1 of *Bamboo mosaic virus* are essential for virus movement. The Journal of General Virology 85, 251–259. 14718640 10.1099/vir.0.19442-0

[CIT0038] Lin MK, Hu CC, Lin NS, Chang BY, Hsu YH 2006 Movement of potexviruses requires species-specific interactions among the cognate triple gene block proteins, as revealed by a trans-complementation assay based on the *Bamboo mosaic virus* satellite RNA-mediated expression system. The Journal of General Virology 87, 1357–1367. 16603539 10.1099/vir.0.81625-0

[CIT0039] Lin NS, Lin FZ, Huang TY, Hsu YH 1992 Genome properties of *Bamboo mosaic virus*. Phytopathology 82, 731–734.

[CIT0040] Liu C, Nelson RS 2013 The cell biology of Tobacco mosaic virus replication and movement. Frontiers in Plant Science 4, 12. 23403525 10.3389/fpls.2013.00012 PMC3568708

[CIT0041] Marmagne A, Rouet MA, Ferro M, Rolland N, Alcon C, Joyard J, Garin J, Barbier-Brygoo H, Ephritikhine G 2004 Identification of new intrinsic proteins in *Arabidopsis* plasma membrane proteome. Molecular & Cellular Proteomics 3, 675–691. 15060130 10.1074/mcp.M400001-MCP200

[CIT0042] Nagasaki-Takeuchi N, Miyano M, Maeshima M 2008 A plasma membrane-associated protein of *Arabidopsis thaliana* AtPCaP1 binds copper ions and changes its higher order structure. Journal of Biochemistry 144, 487–497. 18664522 10.1093/jb/mvn092

[CIT0043] Nagasaki N, Tomioka R, Maeshima M 2008 A hydrophilic cation-binding protein of *Arabidopsis thaliana*, AtPCaP1, is localized to plasma membrane via N-myristoylation and interacts with calmodulin and the phosphatidylinositol phosphates PtdIns(3,4,5)P(3) and PtdIns(3,5)P(2). The FEBS Journal 275, 2267–2282. 18397324 10.1111/j.1742-4658.2008.06379.x

[CIT0044] Niehl A, Heinlein M 2011 Cellular pathways for viral transport through plasmodesmata. Protoplasma 248, 75–99. 21125301 10.1007/s00709-010-0246-1

[CIT0045] Nühse TS, Boller T, Peck SC 2003 A plasma membrane syntaxin is phosphorylated in response to the bacterial elicitor flagellin. The Journal of Biological Chemistry 278, 45248–45254. 12949074 10.1074/jbc.M307443200

[CIT0046] Park MR, Seo JK, Kim KH 2013 Viral and nonviral elements in potexvirus replication and movement and in antiviral responses. Advances in Virus Research 87, 75–112. 23809921 10.1016/B978-0-12-407698-3.00003-X

[CIT0047] Peña EJ, Heinlein M 2012 RNA transport during TMV cell-to-cell movement. Frontiers in Plant Science 3, 193. 22973280 10.3389/fpls.2012.00193 PMC3428586

[CIT0048] Prasanth KR, Huang YW, Liou MR, Wang RY, Hu CC, Tsai CH, Meng M, Lin NS, Hsu YH 2011 Glyceraldehyde 3-phosphate dehydrogenase negatively regulates the replication of *Bamboo mosaic virus* and its associated satellite RNA. Journal of Virology 85, 8829–8840. 21715476 10.1128/JVI.00556-11 PMC3165797

[CIT0049] Prokhnevsky AI, Peremyslov VV, Dolja VV 2005 Actin cytoskeleton is involved in targeting of a viral Hsp70 homolog to the cell periphery. Journal of Virology 79, 14421–14428. 16254376 10.1128/JVI.79.22.14421-14428.2005 PMC1280222

[CIT0050] Qin T, Li J, Yuan M, Mao T 2012 Characterization of the role of calcium in regulating the microtubule-destabilizing activity of MDP25. Plant Signaling & Behavior 7, 708–710. 22751329 10.4161/psb.20336 PMC3583946

[CIT0051] Schäffner AR 1998 Aquaporin function, structure, and expression: are there more surprises to surface in water relations?Planta 204, 131–139. 9487723 10.1007/s004250050239

[CIT0052] Schoelz JE, Harries PA, Nelson RS 2011 Intracellular transport of plant viruses: finding the door out of the cell. Molecular Plant 4, 813–831. 21896501 10.1093/mp/ssr070 PMC3183398

[CIT0053] Scholthof HB 2005 Plant virus transport: motions of functional equivalence. Trends in Plant Science 10, 376–382. 16023398 10.1016/j.tplants.2005.07.002

[CIT0054] Taliansky M, Torrance L, Kalinina NO 2008 Role of plant virus movement proteins. Methods in Molecular Biology 451, 33–54. 18370246 10.1007/978-1-59745-102-4_3

[CIT0055] Tian C, Wan P, Sun S, Li J, Chen M 2004 Genome-wide analysis of the GRAS gene family in rice and *Arabidopsis*. Plant Molecular Biology 54, 519–532. 15316287 10.1023/B:PLAN.0000038256.89809.57

[CIT0056] Tsai CH, Cheng CP, Peng CW, Lin BY, Lin NS, Hsu YH 1999 Sufficient length of a poly(A) tail for the formation of a potential pseudoknot is required for efficient replication of *Bamboo mosaic potexvirus* RNA. Journal of Virology 73, 2703–2709. 10074116 10.1128/jvi.73.4.2703-2709.1999 PMC104026

[CIT0057] Verchot-Lubicz J, Torrance L, Solovyev AG, Morozov SY, Jackson AO, Gilmer D 2010 Varied movement strategies employed by triple gene block-encoding viruses. Molecular Plant-Microbe Interactions 23, 1231–1247. 20831404 10.1094/MPMI-04-10-0086

[CIT0058] Vijayapalani P, Maeshima M, Nagasaki-Takekuchi N, Miller WA 2012 Interaction of the trans-frame potyvirus protein P3N-PIPO with host protein PCaP1 facilitates potyvirus movement. PLoS Pathogens 8, e1002639. 22511869 10.1371/journal.ppat.1002639 PMC3325209

[CIT0059] Wu CH, Lee SC, Wang CW 2011 Viral protein targeting to the cortical endoplasmic reticulum is required for cell-cell spreading in plants. The Journal of Cell Biology 193, 521–535. 21518793 10.1083/jcb.201006023 PMC3087015

[CIT0060] Yeh TY, Lin BY, Chang YC, Hsu YH, Lin NS 1999 A defective RNA associated with *Bamboo mosaic virus* and the possible common mechanisms for RNA recombination in potexviruses. Virus Genes 18, 121–128. 10403698 10.1023/a:1008008400653

[CIT0061] Zhao J, Liu Q, Zhang H, Jia Q, Hong Y, Liu Y 2013 The rubisco small subunit is involved in tobamovirus movement and Tm-2²-mediated extreme resistance. Plant Physiology 161, 374–383. 23148080 10.1104/pp.112.209213 PMC3532268

