# Supplementary material for: Plasma membrane-associated cation-binding protein 1-like protein negatively regulates intercellular movement of BaMV
Source: J Exp Bot. 2017 Aug 29;68(17):4765–74. doi: 10.1093/jxb/erx307 (PMC5853580; doi:10.1093/jxb/erx307)
Supplement: Supplementary_figures_S1_S3 [file erx307_suppl_supplementary_figures_s1_s3.pdf]

```
NtPIP     MGYWQAKVVPKFKQIFDKNGPKKAAAAEACKTFDEAKEQYSKEFEEKKTELQPKVVEIYEAAAVEIKTLVKGPKVSGLKK
AtPCaP1   MGYWNSKVVPKFKKLFEKNSAKKAAAAEATKTFDESKETINKEIEEKKTELQPKVVETYEATSAEVKALVRDPKVAGLKK
VvPCaP1L  MGYWKSKVLPKIKKIFEKSGTKKTAAAEACKSFDDSKEEINKDFEAKKDELQPKVIEIYEASSTEIKTLVKEPKEAGLK-
LjPCaP1L  MGYWKSKVLPKIKKVFEKNGAKKTAAAEAIKSFDESKEEYNKVFEEKKVELQTKVIEIYEASPAEIKSLVKEQKEAGLKK
GmPCaP1   MGYWKSKVLPKIKKVFEKNSTKKAAAAEATKSFDESKEEYNKAFEEKKTELQTKVVEIYEASSTEIKSLVKEPKEAGLKK
NbPCaP1L  MGYWQAKVVPKLKQIFDKNGPKKAAAAEAWKTFDEAKEQYSKEFDEKKTELQPKVVEIYEAAAVEIKTLVKGPKVSGLKK
          1........10........20........30........40........50........60........70.........

NtPIP     HSTQVQKFLDELVKIEFPGSKAVSEASSNFGPSYVSGPVLFVFEKVSTFIVTEDKKEEEA--------AASEAEATTAAP
AtPCaP1   NSAAVQKYLEELVKIEFPGSKAVSEASSSFGAGYVAGPVTFIFEKVSVFLPEEVKTKEIPVEEVKAEEPAKTEEPAKTEG
VvPCaP1L  NSTAVQKFLDELVKIEFPGSKAVCEASSKYGPGLVSGPVLFLFEKVSTFIVTEENEEELP--------PPTTNTTGEEEA
LjPCaP1L  NPTEVHKFLEELAKIEFPGSKVVSEASSKFGPALVPGPVLFVFEKVSTFIVTEEK-VEAP--------PA-ETKTEEEAS
GmPCaP1   NSTEVQKFLEELVKIDFPGSKAASEASSKFGPALASGSVFFVFEKVSTFIVTEEKEVEAP--------PAVETKTEEETS
NbPCaP1L  HSTEVQKFLDELVKIEFPGSKVVSEASSNFGPSYVSGPVLFVFEKVSTLIVTEDKKEEEAAIPVA---AASEAEATTTAP
          81.......90........100.......110.......120.......130.......140.......150........

NtPIP     VEVKEKEIVVEAEAKKEEEPAPAPAATPAA----------------EVAPAEVAP--AADV--APAKVEEAPAAVAA---
AtPCaP1   T-SGEKEEIVE-ETKKGETPETAVVEEKKP----------------EVEEKKEEATPAPAVVETPVKEPETTTTAPV---
VvPCaP1L  S-GKEKEIVIE-EEKKEE---------VAV----------------MVEEREA---------------TEAVA--AA---
LjPCaP1L  T-TKDREIVVE-EEKKEEEKPQVAEETTEKVESEPP----------KEEEKPAEPVVAEAP--PAAKEEEKPV--EPVAE
GmPCaP1   SVVKERETVVE-EEKKEEEKPQADETSDEK----------------KVEEKQAE---------TAAKEEEKPA--EP---
NbPCaP1L  VEVKEKEIVVEAEAKKEEEPAPAPAPAPAPAPAPAPAPAPAAAPAAEVAPAEVAP--AADV--APAKVEEAPAAVAA---
          161......170.......180.......190.......200.......210.......220.......230........

NtPIP     ---PEPPKA
AtPCaP1   ---AEPPKP
VvPCaP1L  ---AEPAKP
LjPCaP1L  VEKTEPPKP
GmPCaP1   ---AEPPKP
NbPCaP1L  ---PEPPKA
          241......
```

**Supplementary Figure S1.** Amino acid sequence alignment of PCaP1 with related proteins from various plants: PCP1 (*Arabidopsis thaliana,* At4g20260), DREPP1 (*Nicotiana tabacum*, O19910), DREPP4 (*N. tabacum*, O24124), Q9SMK5 (*Cicer arietinum*) and Q0JPA6 (*Oryza sativa;* gene name, Os1g0233000). Phosphorylation sites were predicted by using NetPhos 2.0 (http://www.cbs.dtu.dk/services/NetPhos/). The completely conserved residues have no color background, identical residues are yellow, similar residues are cyan and different residues are black.

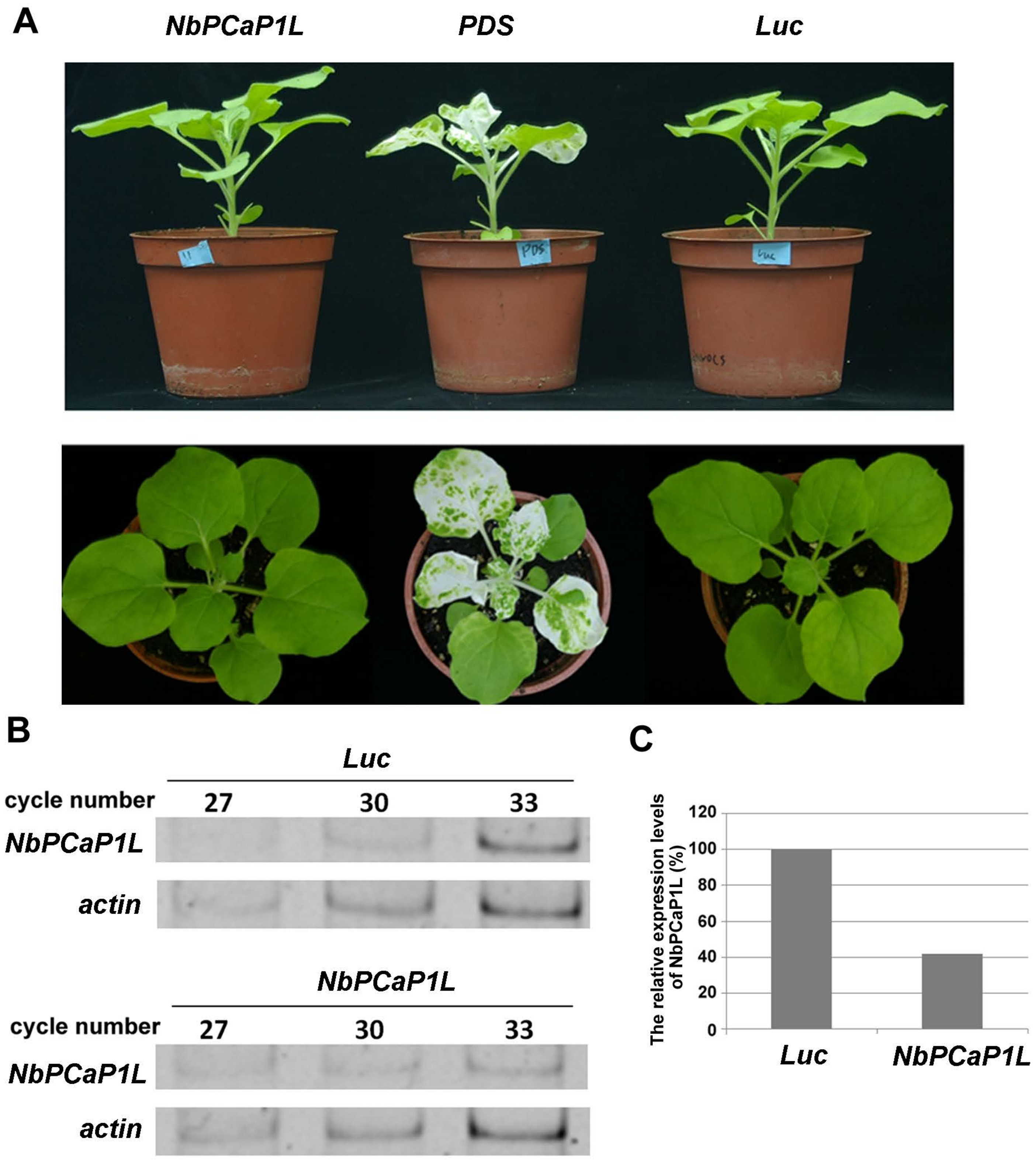


**Supplementary Figure S2.** The morphology of control and *NbPCaP1L* knockdown plants. (A) Morphology of knockdown plants. The positive control is the phytoene desaturase knockdown plant (PDS), whereas the negative control is the luciferase gene knockdown plant (Luc). (B,C) Semi-quantitative RT-PCR of mRNA level of *NbPCaP1L* in Luc and ACGT11-knockdown plants. The cycling number of the RT-PCR is indicated. The expression of β-actin was an internal control.

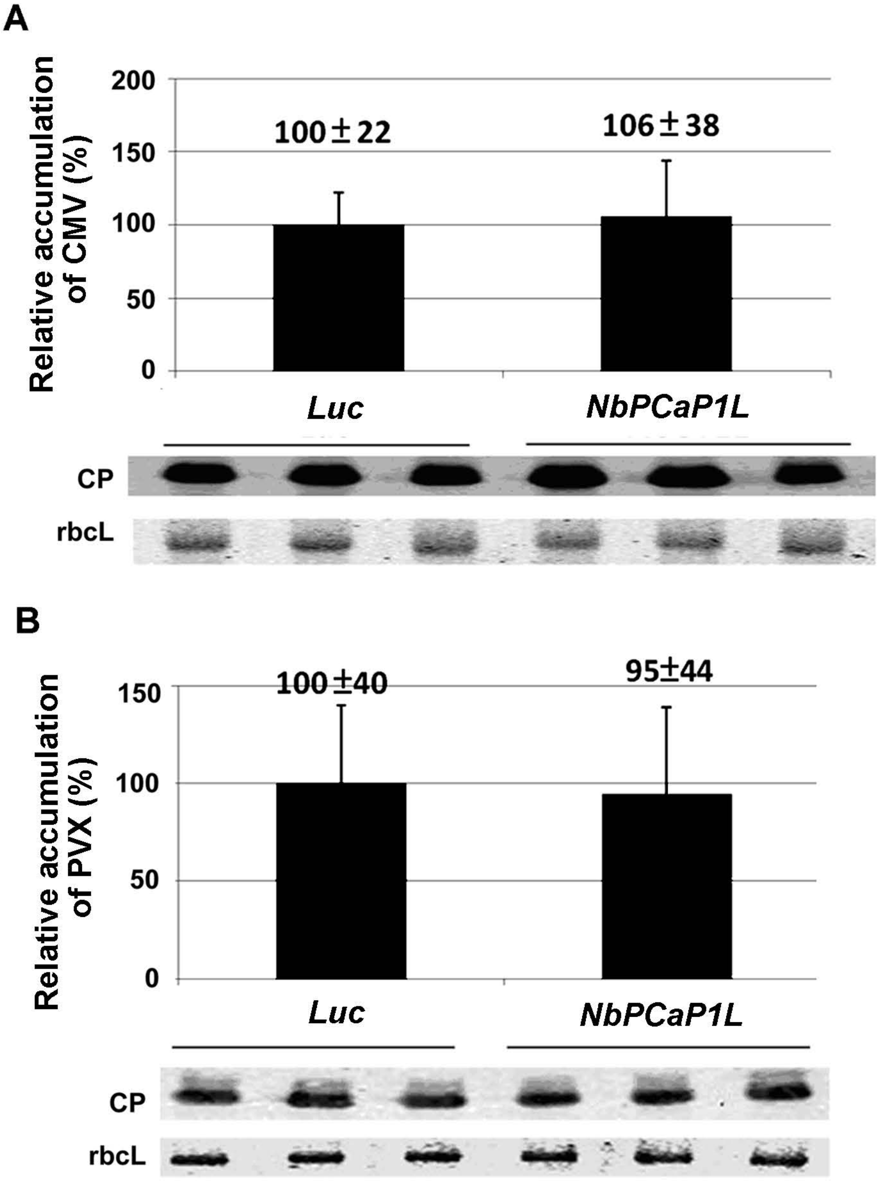


**Supplementary Figure S3.** The relative accumulation of viral coat protein (CP) in *NbPCaP1L*-knockdown and control plants. Western blot analysis of CP accumulation in control and *NbPCaP1L*-knockdown plants inoculated with (A) *Cucumber mosaic virus* (CMV) or (B) *Potato virus X* (PVX) at 5 d post-inoculation. Data are mean±SD relative accumulation of coat protein from at least 3 independent experiments with at least 3 plants in each experiment. *Luc*: luciferase knockdown control plants; *NbPCaP1L*: *NbPCaP1L*-knockdown plants; CP: coat protein; rbcL: RuBisCO large subunit (the loading control for normalization).
